# A Rare Case of Metastatic Rectal Cancer to the Brain Presenting with Headaches and Memory Difficulties

**DOI:** 10.14309/crj.0000000000001178

**Published:** 2023-11-03

**Authors:** Prabhat Kumar, Pearl Aggarwal, Rajat Garg, Amandeep Singh, Talal Adhami

**Affiliations:** 1 Department of Internal Medicine, Cleveland Clinic Foundation, Cleveland, OH; 2 Department of Internal Medicine, University Hospitals, Cleveland, OH; 3 Department of Gastroenterology and Hepatology, Cleveland Clinic Foundation, Cleveland, OH

## CASE REPORT

**Figure 1.**
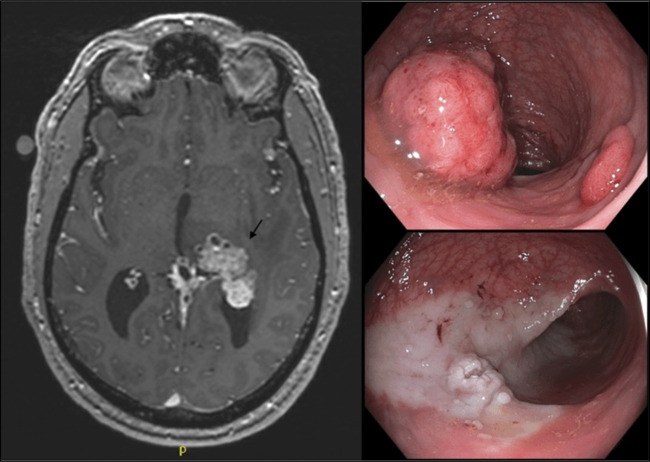
Brain MRI demonstrating metastasis to the left thalamus (black arrow). On the right, colonoscopy images showing a fungating mass in the rectum and postsnare colon. MRI, magnetic resonance imaging.

Colorectal cancer rarely spreads to the brain, but we report a unique case of rectal cancer with brain metastases. A 50-year-old White man presented with chronic headaches, memory difficulties, and occasional rectal bleeding after hard stools. His last colonoscopy was not successful because of poor preparation. Subsequently, he experienced dizziness, facial weakness, and seizures. Brain magnetic resonance imaging revealed metastatic deposits in the left thalamus. Partial tumor resection revealed adenocarcinoma positive for AE1/AE3, CK7, and CDX2, indicating a pancreaticobiliary or gastrointestinal origin. Negative stains for SATB2, CK20, PAX 8, and S100 strengthened this assumption. The Ki-67 proliferative index was 75.0%. Abdominal/pelvic and chest computed tomography scans showed no signs of cancer. A repeat colonoscopy found a nonobstructing 7 × 5 cm pedunculated polypoid rectal lesion (Figure 1). It was successfully removed, and histology confirmed invasive moderately differentiated adenocarcinoma from tubulovillous adenoma without lymphovascular invasion.

Metachronous brain metastasis of colorectal cancer is rare and carries a poor prognosis because chemotherapy does not penetrate the central nervous system.^1^ Our case presents a rare scenario of undetected rectal cancer with isolated brain metastasis and neurological manifestations.^2^ A thorough investigation is advised in similar cases.^3^ Treatment modalities encompass radiation therapy, anti-epidermal growth factor receptor antibody therapies, monoclonal antibody therapy, and chemotherapy.^4^

## DISCLOSURES

Author contributions: P. Kumar: substantial contributions to the conception, acquisition, analysis, and interpretation of data for the work and drafting and reviewing it critically for important intellectual content. P. Aggarwal: substantial contributions to the interpretation of data for the work and reviewing the work critically for important intellectual content. R. Garg: substantial contributions to the conception; drafting the work for important intellectual content; and final approval of the version to be published. A. Singh: substantial contributions to the conception, acquisition, analysis, and interpretation of data for the work and reviewing it critically for important intellectual content. A. Talal: substantial contributions to the analysis of data for the work and drafting the work. All authors gave final approval of the version to be published and agree to be accountable for all aspects of the work in ensuring questions related to the accuracy or integrity of any part of the work are appropriately investigated and resolved. P. Kumar is the article guarantor.

Financial disclosure: None to report.

Previous presentation: Previously presented at the ACG Annual Scientific Meeting, October 2022, Charlotte, North Carolina.

Informed consent was obtained for this case report.
